# European Correspondence

**Published:** 1868

**Authors:** W. S. Armstrong

**Affiliations:** Paris, France


### EUROPEAN CORRESPONDENCE.

PARIS, FRANCE, December 27, 1867.

*To Editors Atlanta Medical and Surgical Journal:*

From those who have had opportunities of observing from year to year, I learn that Paris has not been so crowded with the disciples of Æsculapius, any one season for a long time as during the present; and the correctness of this seems to be well sustained by the great number of foreigners to be met with at the clinics. In fact, at some of the most popular clinics, as at Desmarre's on Diseases of the Eye, and Jarjavy's on Surgery, the foreign element seems to preponderate; and especially is this the case at Dr. Desmarre's. This seems, at least in part, due to the Exhibition, which has drawn so many to the modern Athens during the summer; and now since it is over, they are devoting themselves to study. The United States is not, perhaps, as well represented in numbers as usual. But she can certainly claim what no other nation can, viz: that she has at this time a female student of medicine attending the hospitals of Paris. I first saw her at the Charité, several days ago, among the students who were accompanying one of the surgeons in his morning visit. On this occasion there was a large number present; and whenever this is the case, it is well known how difficult it is to get sufficiently near to hear the remarks of the Professor, and at the same time see the patient. Yet her crinoline (for her dress is that usually worn by ladies) generally sufficed to give her a position at the bed-side of the patient; when, however, that was not sufficiently regarded, her seeming determination to overcome all obstacles, together with her small size, enabled her to glide through the crowd and take position by the side of the surgeon, to which she appeared to feel entitled. The sick, of course, had never heard of a Doctress; consequently, one of the male patients, whose malady required that his person should be more than ordinarily exposed, first inquired what "*petite femme*" (little woman) that was, and what she wanted. After the visit, she went to the amphitheatre to see the operations and to take notes on the lecture. To an American physician standing near me, she spoke very lightly of the female medical schools in the States, especially one in New York, which city she claims as her home. I must do her the justice to say that her manners are genteel, and that she seems earnest in her undertaking.

The case of aneurism treated by digital compression, mentioned in my last, has continued to do well. The large clot contained in the aneurismal sack was removed with a pair of scissors by piece meal, from day to day, as suppuration detached it from the parieties. A compress and bandage over the sack, together with irrigation, constitutes the treatment. In a very short time, the sack will be entirely filled with granulations, and the cure completed. The limb below the knee has been œdematous, but is now normal.

There is always more or less puerperal fever to be found in the hospitals here. But recently there seems to be an epidemic among the lying-in women. Whether this is owing to causes within the hospital, or is attributable to what is vaguely termed epidemic influences, is not apparent. The wards are not more crowded than usual. But one thing is to be observed in all the *old* hospitals here—those that have been built several hundred years—which must operate more or less unfavorably upon the inmates; viz: the want of proper ventilation. There is really no ventilation to be had in them, unless the doors and windows are left open, which is impracticable in this climate during the winter months. It has been long remarked that capital operations do badly within the city; and, indeed, I am thoroughly persuaded that this is the chief difficulty in the way of treating them. On the contrary, it is pleasant to note, that those of more recent construction have been greatly improved in that respect, and that one just finished has all of the modern improvements of ventilation introduced in it.

But to resume. This epidemic of puerperal fever does not present that regularity in its march, which is usually observed, for in many of the cases pleuritis and pericarditis have been prominent symptoms. Yet, notwithstanding the addition of one or both of these complications, the majority of the cases recover. In a *post mortem* examination seen recently, pus was found in the substance of uterus and in the broad ligament of the left side, but in no other organ. Newly-formed adhesions were formed in the illiac fossæ on both sides, also in the thoracic cavity, on both sides, and in the pericardium. The pleuritic symptoms are generally confined to one side, and it has been noticed that when the left is affected, the gravity of the case is increased.

Within the last few years, the diagnosis of affections of the larynx has been rendered as certain as in any part of external pathology. What has heretofore been uncertain in this respect, for want of proper appliances to expose this organ, has been entirely remedied by the introduction of the laryngoscope. By its use, it is quite as easy to expose to view the superior cavity of the larynx and vocal cords, and to apply remedies to them, as it is to expose and treat diseases of the fauces. A skillful surgeon may even expose to view more or less of the trachea.

M. Fauvel has perhaps given this subject more attention than any one here. He succeeds better in exposing the larynx to a number than any one I have yet seen. To effect this he uses the ordinary Drummond light, the rays of which pass through a brass tube five feet in length, to the mouth of the patient, who sits in front. By taking position on each side of this instrument, quite a number are enabled to see the vocal cords, &c., at the moment the reflector is properly adjusted. Among a large number of cases treated, I have watched the progress of two with much interest. The first, in an old man of sixty years, is a tumor situated on the left side of the larynx, in front of the vocal cord of that side. The pressure which it exerts on the surrounding parts, has so interfered with the function of the organ as to produce complete aphonia, and at times difficulty of respiration. To attempt an operation with cutting instruments, to say nothing of hemorrhage, in an organ so sensitive to the touch, would be very questionable. Hence the removal of the tumor has been commenced by the use of galvano-caustic, an admirable method of using the actual cautery. A portion of the tumor has already been removed. Its entire ablation will require time. But there is every reason to expect a perfect cure, as he has already received partial relief.

The second case is one of paralysis of the left vocal cord. This affection is specific in its origin. Yet it was determined to try the effect of electricity in the commencement. It was persevered in for several weeks, but while it stimulated the cord to contraction during the application, no permanent good resulted. The hoarseness of the voice, and the red, conjested state of the cord seemed more marked than at the beginning of the treatment. Iodide of potassium was next employed. The hoarseness and redness of the vocal cord began to disappear in a few days. The paralysis has also gradually yielded, thereby establishing the correctness of the diagnosis.

I have met with your corresdondent, Dr. L. P. Yandall, of Louisville, Kentucky, every day for the last week. He is genial and warm hearted, and a gentleman of fine culture. I have regretted that our studies have prevented us from meeting oftener. He has been devoting himself almost exclusively to affections of the skin, both in London and this city.

W. S. ARMSTRONG, M. D.

